# Efficacy of a single dose switch from aflibercept to ranibizumab in the treatment of neovascular age-related macular degeneration

**DOI:** 10.1371/journal.pone.0342927

**Published:** 2026-03-06

**Authors:** Johannes Iby, Leonard Coulibaly, Marlene Hollaus, Lusine Yeghiazaryan, Ursula Schmidt-Erfurth, Wolf Bühl, Stefan Sacu

**Affiliations:** 1 Department of Ophthalmology and Optometry, Medical University of Vienna, Vienna, Austria; 2 Christian Doppler Laboratory for Ophthalmic Image Analysis, Department of Ophthalmology and Optometry, Medical University of Vienna, Vienna, Austria; 3 Institute of Medical Statistics, Center for Medical Data Science, Medical University of Vienna, Vienna, Austria; Akita University: Akita Daigaku, JAPAN

## Abstract

**Purpose:**

To analyze morphological and functional response after administration of a single dose of ranibizumab in eyes under treat&extend aflibercept in neovascular age related macular degeneration (nAMD). Additionally, we aimed to identify predictive factors in anti-vascular endothelial growth factor (VEGF) treatment.

**Methods:**

A total of 115 patients (133 eyes; 34 male, 81 female, m:f = 1:2.4) with a mean age of 79.9 ± 8.3 years at baseline (BSL) were included in this retrospective analysis. All eyes had received aflibercept treatment for nAMD before BSL and were subsequently administered a single dose of intravitreal ranibizumab. Data of two visits pre and two visits after BSL were evaluated and compared. Primary outcome measure was the mean change in central macular thickness (CMT, µm). CMT-changes between BSL and the next follow-up visit (V4) were roughly divided into decrease (<−10µm), equal (−10 to +10 µm) or increase (> +10µm). We also assessed changes in best-corrected visual acuity (BCVA) and identified influencing factors on CMT and BCVA using linear mixed models (LMM).

**Results:**

No significant differences in CMT and BCVA were observed over the visits (p > 0.05). Factors influencing CMT included prior anti-VEGF injections (p < 0.001), the time interval between BSL and visit 4 (p < 0.001), and patient age (p = 0.032). The number of prior anti-VEGF injections significantly influenced BCVA (p = 0.003). After a single administration of ranibizumab, in 19.5% (n = 26) of eyes CMT decreased, in 21.8% of eyes (n = 29) CMT increased and in 36.8% of eyes (n = 49) it remained equal.

**Conclusion:**

Our study indicates that changes in CMT are affected by age, the number of previous injections, and the time interval between visits. These biomarkers may offer valuable insights for future research on switching anti-VEGF drugs to optimize treatment strategies for nAMD. A single dose treatment switch to ranibizumab yielded non-inferior outcomes.

## Introduction

Age-related macular degeneration (AMD) is recognized as “the leading cause of legal blindness” in developed countries [1]. It affects almost 10% of people > 65 years of age and 25% of those > 75 years of age [2]. AMD can be broadly categorized into two subtypes: the non-neovascular form and the neovascular variant (nAMD). Recently, the first treatment options for non-neovascular AMD have been approved in the United States, although they have yet to reach the masses [3,4].

Vascular endothelial growth factor (VEGF) plays a central role in the development of neovascular age-related macular degeneration (nAMD) and remains the primary target for therapeutic intervention. Anti-VEGF agents have shown significant efficacy by restoring retinal morphology, improving and preserving neurosensory function, and reducing the likelihood of recurrence [1,3]. The introduction of these therapies represents a major milestone in retinal disease management, leading to marked reductions in both legal blindness and visual impairment associated with nAMD [5–7]. Currently, several anti-VEGF agents are available for clinical use, including bevacizumab (Avastin®; Roche Pharma AG), ranibizumab (Lucentis®; Novartis AG), aflibercept (Eylea®; Bayer AG), and most recently brolucizumab (Beovu®; Novartis AG), faricimab (Vabysmo®, Roche Pharma AG) and aflibercept 8 mg (Eylea HD®; Bayer AG). These medications can be administered using various treatment strategies such as pro re nata (PRN), treat and extend (TAE), and observe and plan (OAP) [8]. The choice of anti-VEGF agent and treatment strategy is ultimately determined by the treating physician.

With the global increase in life expectancy, the incidence of AMD is rising, leading to a corresponding increase in the number of monthly anti-VEGF injections required. Increasing treatment intervals with longer-acting anti-VEGF medications, like aflibercept 8 mg, is one way to counteract this treatment burden. Simultaneously, the distribution of ranibizumab via a “port delivery system” (PDS) has recently been introduced in clinical studies and has already been approved in the US. It is essentially a refillable reservoir for anti-VEGF medication implanted in the temporal quadrant of the sclera, allowing for a controlled and sustained delivery of ranibizumab [9,10]. This innovative, long-acting drug delivery system aims to eliminate the need for monthly anti-VEGF injections. The majority of clinical trials seek to identify a response to ranibizumab treatment through intravitreal injection prior to PDS device implantation [10]. Therefore, it is pivotal to show non inferiority when switching from one drug to another.

In the past, clinicians have sought to optimize treatment outcomes by transitioning from one anti-VEGF drug to another [1]. Some patients may not respond adequately to the initially administered anti-VEGF agent and may benefit from switching to an alternative drug [11]. The diminished response observed in eyes undergoing treatment for macular neovascularization (MNV) may be attributed to tachyphylaxis [12]. However, there are limited data on the potential benefits of switching between two anti-VEGF drugs with only a single administration. Furthermore, it remains unclear which medication is the most beneficial when switching, as most studies have reported a transition from bevacizumab and ranibizumab to aflibercept [11,13]. This preference may largely stem from the more recent FDA approval of aflibercept compared to the other agents mentioned.

### Aim

Our aim was to analyze whether a significant difference in morphological response can be observed after administering a single dose of ranibizumab in eyes previously treated with aflibercept.

## Methods

In this retrospective, observational study, all patients were recruited at the Medical University of Vienna. Prior to inclusion, all participants provided written informed consent (Vienna Imaging Biomarker Eye-Study Register, VIBES) after receiving an in-depth explanation of the study. The study was approved by the institutional review board (EK2094/2018) and adhered to the tenets of the Declaration of Helsinki. Data analysis included SS-OCT data as well as best-corrected visual acuity (BCVA, in Snellen) and demographic information. Patients diagnosed with macular neovascularization (MNV) in the context of nAMD who had previously received at least one aflibercept injection were included. Data was accessed multiple times from January to February 2023. Patient data was handled anonymously by all contributors to this study.

All patients diagnosed with nAMD received a single intravitreal injection of ranibizumab at baseline (BSL) after prior treatment with aflibercept. All ranibizumab injections were administered in January 2021. Subsequent treatment following BSL consisted of either aflibercept or ranibizumab.

The date of the single-dose ranibizumab injection was designated as BSL. Data from two visits preceding BSL (V1 and V2) and two visits following BSL (V4and V5), if available, were assessed and compared. The primary outcome measure was the mean change in central macular thickness (CMT, in µm), which served as a metric for evaluating the impact of the treatment transition. CMT data were obtained via SS-OCT automatic segmentation using the Volume Scan protocol of the Cirrus-OCT system (Carl Zeiss Meditec, Dublin, CA). CMT measurements were obtained by positioning the macular thickness map over the fovea and collecting data from the central millimeter. Manual segmentation correction was performed if the retinal layers were not properly aligned.

ChatGPT (GPT-5, OpenAI, San Francisco, CA, USA) was used exclusively for grammar checking and language editing during manuscript preparation. No AI tool was used for data analysis, interpretation, or the generation of scientific content. All suggested edits were reviewed and verified by the authors to ensure accuracy and integrity of the text.

### Statistical analysis

After checking the data for normal distribution, paired t-tests were conducted to determine whether significant differences were present between visits. Changes in CMT between BSL and the next follow-up visit (V4) were roughly divided into decrease (<−10µm), equal (−10 to +10µm) or increase (> +10µm). Linear mixed models (LMM) were then employed to assess whether the number of previously administered injections, age, or sex had a significant influence on the CMT and BCVA differences between BSL and V4. In these models, the respective outcome variables were included as dependent variables, namely the difference in BCVA and CMT between BSL and V4. The number of previous injections, age, and sex were defined as fixed effects, with patient ID considered as a random intercept. All statistical analyses were performed using R Version 4.0.3 (R Foundation for statistical computing, Vienna, Austria). The level of significance was set at α = 0.05.

## Results

**Fig 1:**
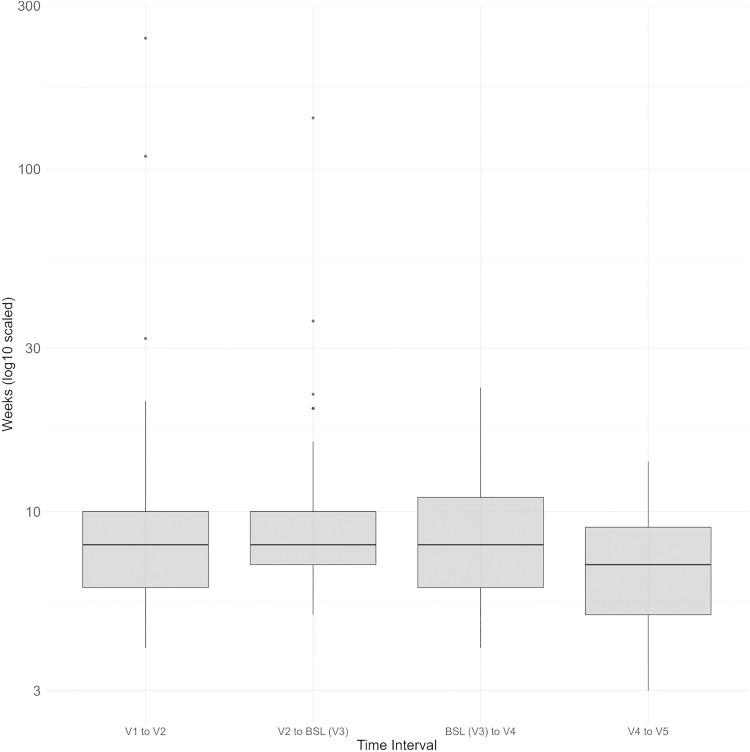
Time interval between the respective visits for all patients in weeks.

One hundred and thirty-three eyes (67 right eyes, 66 left eyes) from 115 patients (34 male, 81 female, m:f = 1:2.4) with a mean age of 79.9 ± 8.3 years at BSL were included in this study. Of the 115 patients, 112 (97.4%) were Caucasian, while 3 (n = 2.6%) were of middle eastern descent. All eyes had previously been diagnosed with nAMD and had received aflibercept before BSL. At BSL, all eyes were treated with ranibizumab. The mean number of injections before BSL was 18.75 ± 14.94, resulting in a treatment switch at BSL for 100% of eyes (n = 133) from aflibercept to ranibizumab. Treatment after the switch varied between patients. After a single dose of ranibizumab, 87.2% (n = 116) of eyes continued treatment with aflibercept and 4.5% (n = 6) with ranibizumab. Four eyes required no further treatment. Details for the subgroups, demographic data as well as mean CMT and BCVA per visit are presented in Table 1. At BSL, 28.6% (n = 38) of eyes showed signs of crystalline lens opacification, 2.3% (n = 3) had a clear phakic lens, and 57.1% (n = 76) were pseudophakic. No data was available for 12% of eyes (n = 16). To evaluate differences in changes in CMT and BCVA between consecutive pairs of visits (V2 - V1, …, V5 - V4), paired t-tests were calculated. Only the BCVA reduction from V1 to V2 was marginally significant (est.: −0.029; p = 0.047, Table 2). Furthermore, the time interval between each pair of visits was correlated with changes in CMT and BCVA. For the difference between BSL and V4, a correlation between change in CMT and length of the interval was identified (est.: 0.243, p: 0.018). From BSL to V4, 19.5% of eyes (n = 26) showed a decrease in CMT, 21.8% (n = 29) an increase, and 36.8% (n = 49) remained stable. Data were unavailable for 21.8% of eyes (n = 29). Details for the respective interval lengths are shown in Fig 1.

**Table 1:** Demographic and clinical data for the general study population as well as different subgroups switching between aflibercept and ranibizumab at BSL (V3).

	Overall (n = 133)	Aflibercept-Ranibizumab- Aflibercept (n = 116)	Aflibercept-Ranibizumab-Ranibizumab (n = 6)	Aflibercept-Ranibizumab- None (n = 4)	Aflibercept-Ranibizumab- Missing (n = 7)
**Age (y)**	79.92 ± 8.27	79.33 ± 8.46	81.5 ± 5.2	83.86 ± 4.6	86.25 ± 7.76
**Sex:**					
**Male**	34 (29.6%)	28 (28%)	1 (25%)	1 (25%)	4 (57.1%)
**Female**	81 (70.4%)	72 (72%)	3 (75%)	3 (75%)	3 (42.9%)
**Previous anti-VEGF injections**	18.75 ± 14.94	19.97 ± 15.46	9.83 ± 5.78	12.5 ± 10.66	9.86 ± 4.41
**CMT** mean±SD median [IQR]					
**V1**	270 ± 61 263.5 [234–308.25]	269 ± 62 263.5 [229.25–308.75]	234 ± 49 228 [208.5–257]	282 ± 67 263 [236–308.75]	286 ± 57 264 [251.5–298]
**V2**	274 ± 76 270 [227–301]	271 ± 66 263 [226–301]	298 ± 137 258 [233–284]	348 ± 193 266.5 [240.5–375]	266 ± 52 275 [252–295.5]
**BSL (V3)**	274 ± 80 259.5 [226–314.75]	270 ± 72 256 [226.5–304]	325 ± 134 300.5 [239.25–358.75]	322 ± 144 268.5 [239–351.5]	261 ± 85 270 [214.5–299]
**V4**	271 ± 93 260.5 [226–301.75]	275 ± 91 261 [226–307]	244 ± 40 248 [219–272.75]	NA	NA
**V5**	279 ± 88 260 [219–318]	274 ± 86 259.5 [217.5–317.75]	376 ± 53 406 [360.5–406]	NA	NA
**BCVA** mean±SD median [IQR]					
**V1**	0.44 ± 0.29 0.4 [0.19–0.63]	0.45 ± 0.29 0.4 [0.25–0.63]	0.36 ± 0.22 0.32 [0.32–0.5]	0.21 ± 0.29 0.1 [0.01–0.31]	0.5 ± 0.42 0.4 [0.22–0.6]
**V2**	0.42 ± 0.28 0.4 [0.1–0.63]	0.44 ± 0.29 0.4 [0.25–0.63]	0.41 ± 0.18 0.45 [0.34–0.5]	0.15 ± 0.23 0.06 [0.01–0.2]	0.29 ± 0.28 0.17 [0.07–0.54]
**BSL (V3)**	0.43 ± 0.27 0.4 [0.25–0.63]	0.45 ± 0.27 0.4 [0.25–0.63]	0.34 ± 0.2 0.41 [0.16–0.5]	0.15 ± 0.23 0.06 [0.01–0.2]	0.37 ± 0.27 0.4 [0.18–0.5]
**V4**	0.43 ± 0.28 0.4 [0.14–0.63]	0.44 ± 0.28 0.4 [0.25–0.63]	0.3 ± 0.19 0.32 [0.14–0.47]	NA	NA
**V5**	0.49 ± 0.3 0.5 [0.27–0.63]	0.49 ± 0.3 0.5 [0.28–0.63]	0.37 ± 0.23 0.5 [0.3–0.5]	NA	NA
**Interval** (weeks)					
**V1-V2**	11.17 ± 22.4 8 [6–10]	9.39 ± 10.19 8 [6–10]	7.33 ± 2.42 7 [6–9.5]	9 ± 4.16 9 [7–11]	44.57 ± 86.75 12 [9–17.5]
**V2-BSL (V3)**	10.32 ± 12.04 8 [7–10]	10.15 ± 12.79 8 [6–10]	10.17 ± 2.79 11.5 [9.5–12]	12.25 ± 6.85 10 [7.75–14.5]	12.14 ± 4.1 12 [10–13]
**BSL (V3) −4**	8.56 ± 3.49 8 [6–11]	8.56 ± 3.46 8 [6–10.5]	8.67 ± 4.46 8.5 [5–12]	NA	NA
**V4-V5**	7.17 ± 2.62 7 [5–9]	7.28 ± 2.58 7 [6–9]	4.55 ± 2.31 3 [3–5]	NA	NA

Values are presented as mean ± standard deviation and/or median [IQR]. BCVA = best corrected visual acuity, CMT = central maular thickness, V1 = visit 1, V2 = visit 2, V4 = visit 4, V5 = visit 5.

**Table 2:** Mean changes in central macular thickness (CMT) and best corrected visual acuity (BCVA) over the study visits.

Time interval	V1 to V2	V2 to BSL (V3)	BSL (V3) to V4	V4 to V5
**FUP (weeks)**				
mean ± SD median [IQR]	11.17 ± 22.40 8 [6–10]	10.32 ± 12.04 8 [7–10]	8.56 ± 3.49 8 [6–11]	7.17 ± 2.62 7 [5–9]
**CMT change**				
mean ± SD Paired t-test (p)	−0.23 ± 49.1 (0.96)	−0.6 ± 49.16 (0.9)	7.48 ± 78.9 (0.36)	−6.88 ± 72.34 (0.51)
**BCVA change**				
Paired t-test (p)	−0.03 ± 0.15 (0.047)	0.004 ± 0.13 (0.740)	−0.005 ± 0.14 (0.738)	0.02 ± 0.1 (0.124)

Values are presented as mean ± standard deviation and/or median [IQR]. BCVA = best-corrected visual acuity, FUP = follow up, V1 = visit 1, V2 = visit 2, BSL (V3) = baseline visit, V4 = visit 4, V5 = visit 5.

### Influences on CMT

**Fig 2:**
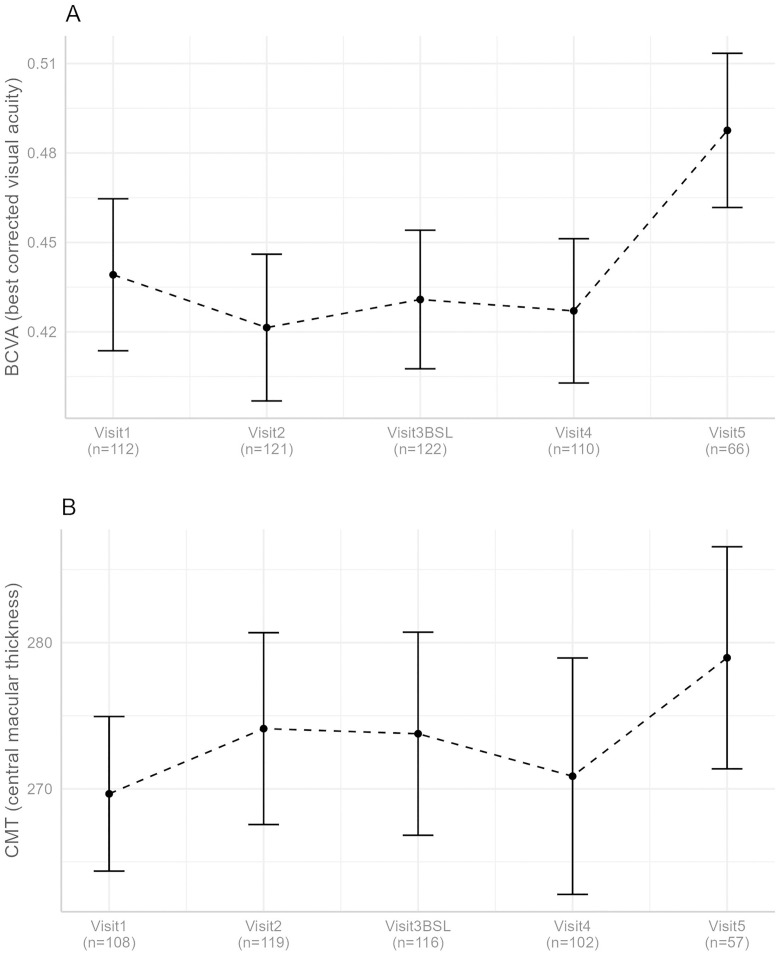
Time course of BCVA (A) and CMT values (B), averaged per time point (+/- standard error).

In the LMM for the change of CMT from BSL to V4, the covariates – number of previous injections (est.: −0.97; p < 0.001), time interval between BSL and V4 (est.: 4.7; p < 0.001), and patient age in years (est.: 2.22; p = 0.032) – had a significant influence on CMT change. Specifically, the longer the interval between BSL and V4, the smaller the reduction in CMT. Additionally, for each unit increase in age, the reduction of CMT from BSL to V4 decreased by 2.2µm. Moreover, a larger number of previously received injections was associated with a stronger reduction of CMT from BSL to V4. Further details are provided in Table 3 and are illustrated in Fig 2.

**Table 3:** Significant results of the linear mixed model for the dependent variables difference in CMT as well as difference in BCVA between BSL and V4.

CMT	Estimate	95% − CI: LL–UL	p
Interval V1 to V3 (BSL)	−0.42	−1.73–0.34	0.28
Interval BSL to V4	4.68	21.97–7.39	**< 0.001**
Injections prior	−0.97	−1.33– − 0.61	**< 0.001**
Age in years	2.22	0.23–4.2	**0.032**
Male vs. female	7.35	−28.93–43.64	0.692
**BCVA**			
Interval V1 to V3 (BSL)	−0.0004	−0.002–0.0009	0.545
Interval BSL to V4	−0.002	−0.009–0.0048	0.546
Injections prior	0.002	0.0007–0.0036	**0.003**
Age in years	−0.0001	−0.0034–0.0031	0.933
Male vs. female	−0.003	−0.06–0.056	0.918

BCVA = best corrected visual acuity, BSL = Baseline, CI = confidence interval, CMT = central macular thickness, Est. = Estimate, V4 = visit 4, 95% − CI: LL- UL: the lower (LL) and upper limits (UL) of 95% − confidence intervals.

### Influences on BCVA

Once more, a linear mixed model was employed to assess whether any of the covariates had a significant influence on BCVA after switching to ranibizumab. Details are provided in Table 3 and are illustrated in Fig 2. Age, sex, and time intervals did not exhibit any significant influences on BCVA. However, the LMM (est.: 0.002; p = 0.003) indicated that a higher number of previously administered injections was associated with a smaller reduction of BCVA from BSL to V4.

## Discussion

This analysis evaluated changes in CMT and BCVA following a single-dose switch from aflibercept to ranibizumab in patients with nAMD. Apart from a marginally significant BCVA decrease from V1 to V2, no significant changes in BCVA and CMT were observed between consecutive study visits, especially after the switch to ranibizumab, suggesting that a single treatment switch does not lead to inferior outcomes. Notably, we did find that the number of previous anti-VEGF injections had a significant positive impact on BCVA changes between BSL and V4. Additionally, we observed that the number of prior injections, the time interval between injections, and patients’ age significantly affected CMT. Older age was associated with less CMT reduction, potentially due to longer disease duration, formation of permanent retinal fluid compartments, or patient preference for extended treatment intervals. Extended intervals between injections also corresponded to higher CMT, consistent with the symptomatic nature of anti-VEGF therapy and the reappearance of sub- and intraretinal fluid.

Comparisons with previous studies highlight these findings. Salcedo et al. reported a significant reduction in CMT after a single-dose switch from bevacizumab to either ranibizumab or aflibercept, but their analysis did not account for the time intervals between visits, which may influence fluid accumulation [11]. Despite also using real-world data, we were unable to replicate their results. Conversely, Waizel et al. observed an initial increase in CMT from 396µm to 499µm after switching from intravitreal bevacizumab to ranibizumab, but CMT returned to 394 µm at the final follow up visit [14]. A Cochrane review on nAMD treatment with anti-VEGF medication reported a mean change in CMT of −165.5 µm over a treatment period of one year from baseline [15].

The absence of significant changes in CMT following the treatment switch is likely multifactorial. Most eyes in this cohort had received extensive anti-VEGF therapy prior to switching and were already maintained on individualized treatment intervals optimized to prevent recurrent exudation. In this context, substantial short-term anatomical changes would not be expected. Moreover, these findings are consistent with the concept of a therapeutic ceiling effect in anti-VEGF therapy, whereby VEGF signaling may already be maximally suppressed and receptor occupancy largely saturated. Given the shared core mechanism of action across anti-VEGF agents, further anatomical improvement may not be achievable despite switching to a molecule with higher binding affinity [16]. It has also been shown that higher baseline visual acuity is strongly associated with smaller gains in BCVA after anti-VEGF therapy, since these eyes have limited potential for improvement [17]. This may partly explain the stability of both BCVA and CMT observed in our cohort. Although aflibercept demonstrates higher affinity for VEGF-A and broader ligand binding compared with other agents, its clinical efficacy appears to plateau at currently approved doses, resulting in comparable functional and anatomical outcomes. Collectively, these observations suggest that in chronically treated eyes with established treatment regimens, switching anti-VEGF agents may primarily serve to maintain disease control rather than induce further improvements in retinal anatomy or visual function. Barthelmes et al. discovered a reduction in active MNV lesions with a treatment switch over an extended period, eventually leading to longer treatment intervals [13]. The time interval between injections directly impacted CMT. While regular anti-VEGF application helps preserve the integrity of retinal layers over time, it remains a symptomatic treatment. Thus, it is not surprising that with extended treatment intervals, CMT increases again due to the reappearance of subretinal and (especially) intraretinal fluid [18].

Across the five study visits, no significant differences in BCVA were observed. Our findings regarding BCVA align with previous studies evaluating switches between aflibercept and ranibizumab. Barthelmes et al. also reported no significant differences in BCVA when switching between aflibercept and ranibizumab over a 12-month period [13]. They noted a gain of 10 or more letters in 10% of eyes, but also a loss of 10 or more letters in 13% of eyes. Although aflibercept was initially expected to provide improved stabilization of visual acuity due to its higher binding affinity and longer intraocular half-life compared to ranibizumab, most studies indicate that while aflibercept effectively reduces CMT, BCVA remains largely unchanged [19,20].

The PDS, developed for use with ranibizumab, is intended for patients with inadequate treatment response or frequent injection requirements. Therefore, it is possible that more patients may switch back to ranibizumab in the near future [10]. As ranibizumab predates aflibercept, most prior studies investigated switches from ranibizumab to aflibercept, but rarely the reverse scenario. In this context, it is important to show that CMT decreased or remained stable in 56.3% of eyes when switching back to ranibizumab for a single dose, with increases in CMT only noted in 21.8% of eyes (n = 29).

Among factors evaluated in the LMM, the number of anti-VEGF injections prior to BSL had significant effects on BCVA. Specifically, the higher the number of previously received injections, the weaker the reduction of BCVA between BSL and V4. It can be inferred that eyes already receiving multiple anti-VEGF injections were treated over an extended period. This observation aligns with studies showing that long-term anti-VEGF therapy may prevent vision loss but is often accompanied by gradual declines in BCVA due to fibrosis and atrophy [20,21].

Evaluating the effect of switching between anti-VEGF agents presents several challenges. As mentioned previously, many of the eyes included in the study had already undergone prolonged treatment for a long-standing disease, potentially resulting in fibrosis and atrophy formation, leading to a poor visual outcome despite favorable anatomical response. Evaluating the effect of a single dose treatment switch without a control group is challenging, as subtle changes may only become apparent when compared to a control group with an equal treatment history. Moreover, this study relied on real-world data, where retinal characteristics are less uniform than in controlled clinical trial populations. As a retrospective observational analysis, inherent limitations must be acknowledged. No data on potential influencing factors on disease pathogenesis such as obesity, hypertension, smoking status or family history were collected. Nonetheless, our aim was to capture outcomes that more accurately reflect routine clinical practice in a real-world population, where these factors are inherently present. In terms of efficacy, all anti-VEGF drugs ultimately have the same goals – to restore retinal morphology with minimal treatment burden. The VIEW 1 and 2 study compared aflibercept and ranibizumab, finding no inferiority between the medications. Both drugs were equally effective in improving visual acuity, although patients receiving aflibercept required fewer injections [22]. Likewise, a study comparing bevacizumab and ranibizumab reached the same conclusion, although slightly more adverse events occurred in patients treated with bevacizumab compared to ranibizumab [23]. As noted by Salcedo et al., it is still unclear whether switching drugs may be beneficial in certain cases, and if so, what the optimal timepoint or drug might be [11]. Therefore, further research on this subject will be key for future treatment individualization, particularly identifying biomarkers predictive of switch benefit. Recent scientific advances have made it possible for aflibercept to be administered in a 4-fold higher concentration [24]. This concept of further developing medications with already proven efficacy may become an interesting topic of future research.

In summary, switching a single aflibercept dose to ranibizumab did not result in inferior outcomes in eyes affected by nAMD. CMT changes were influenced by age, the number of previous injections., and injection intervals. These biomarkers may offer valuable insights for future research on switching anti-VEGF drugs. Further research should clarify which patients benefit most from drug switching and explore optimized dosing strategies to improve long-term visual outcomes.

## Supporting information

S1 File Dataset. (XLSX)
